# Systematic review and meta-analysis protocol of impact of pharmacist-led antibiotic stewardship audit-feedback intervention

**DOI:** 10.1016/j.mex.2025.103399

**Published:** 2025-05-30

**Authors:** Duaa Salem Jawhar, Amer Hayat Khan, Khurshid Alam

**Affiliations:** a School of Pharmaceutical Sciences, Universiti Sains Malaysia, Penang, Malaysia; b Saqr Hospital, Pharmacy Department, Emirates Health Service, Ras al Khaimah, United Arab Emirates

**Keywords:** Antibiotic, Stewardship, Intervention, Feedback, Pharmacist

## Abstract

The rise of resistance to antibiotic agents continues to present a substantial challenge to healthcare systems. In response, antibiotic stewardship programs (ASPs) have been widely endorsed as a core strategy to promote the responsible use of antimicrobials and to curb the progression of antimicrobial resistance (AMR). Pharmacist-led audit and feedback interventions hold significant promise due to pharmacists' clinical expertise and direct role in optimizing antimicrobial prescribing. However, despite their potential, the current body of evidence evaluating the effectiveness of pharmacist-led audit and feedback within ASPs remains limited and fragmented. A clearer understanding of their impact is essential to inform policy decisions, support broader implementation, and enhance the overall effectiveness of stewardship efforts. This protocol has been registered with the PROSPERO under the registration number CRD420251036088. The review will be conducted in accordance with the PRISMA guidelines.A comprehensive search will be carried out across four major databases, and studies will be selected based on clearly defined inclusion and exclusion criteria. The risk of bias in the included studies will be assessed using Cochrane riak of bias assessment tools. Publication bias will be examined using funnel plots and Egger’s test. For the quantitative synthesis, a random-effects model will be employed.

• This protocol outlines a rigorous plan for conducting a high-quality meta-analysis examining the impact of pharmacist-led audit and feedback interventions within antibiotic stewardship programs.
• It details the full methodological framework, from topic selection through to the statistical approaches planned for data synthesis.
• By publicly sharing our protocol through an academic publishing platform, we aim to promote transparency, invite constructive input, and contribute to raising the methodological standards for meta-analyses in the domain of antibiotic stewardship.

## Graphical Abstract

**Figure:**
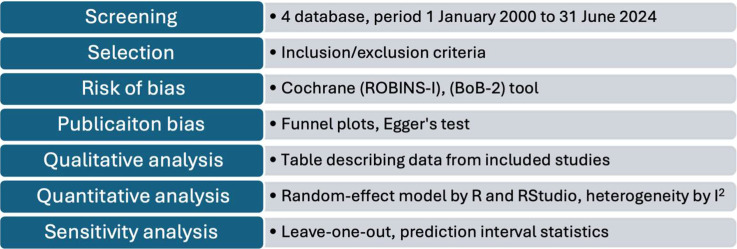

Specifications table

**Table:** 

Subject area:	Pharmacology, Toxicology and Pharmaceutical Science
More specific subject area:	*Antibiotic stewardship*
Name of your protocol:	Systematic review and meta-analysis protocol of impact of Pharmacist-Led Antibiotic Stewardship Audit-Feedback Intervention; Study Protocol
Reagents/tools:	Cochrane (ROBINS-I), (BoB-2) tool R version (4.4.1), RStudio version (2024.09.0+375) EndNote 21 Microsoft Excel version (16.95.1)
Experimental design:	Systematic literature review and meta-analysis following Preferred Reporting Items for Systematic Reviews and Meta-Analyses (PRISMA) guideline.
Trial registration:	This systematic review protocol was registered in the International Prospective Register of Systematic Reviews (PROSPERO) under the registration number: CRD420251036088
Ethics:	This work does not involve direct contact with human subjects, animal experiments or data collection from social media; thus, informed consent or ethical approval not required.
Value of the Protocol:	• This protocol outlines a rigorous plan for conducting a high-quality meta-analysis examining the impact of pharmacist-led audit and feedback interventions within antibiotic stewardship programs. • It details the full methodological framework, from topic selection through to the statistical approaches planned for data synthesis. • By publicly sharing our protocol through an academic publishing platform, we aim to promote transparency, invite constructive input, and contribute to raising the methodological standards for meta-analyses in the domain of antibiotic stewardship.

## Background

Antimicrobial resistance (AMR) has emerged as one of the most critical global health threats of the 21st century, with far-reaching implications for public health, economic development, and the sustainability of modern medical practices. The rise of resistant pathogens compromises the efficacy of essential treatments, leading to prolonged illnesses, increased healthcare costs, and elevated mortality rates. In 2021 alone, AMR was associated with an estimated 4.71 million deaths worldwide, underscoring the urgent need for coordinated global action. Projections indicate that if current trends persist, AMR could contribute to approximately 169 million deaths between 2025 and 2050, potentially reversing decades of progress in infectious disease control and threatening the effectiveness of routine surgical procedures, cancer therapies, and critical care interventions [6,7].

Antibiotic stewardship programs (ASPs) are recognized as one of the cornerstone strategies in the global effort to combat AMR, as they are designed to promote the judicious use of antibiotics through evidence-based prescribing practices. These programs aim to optimize clinical outcomes while minimizing the unintended consequences of antimicrobial use, including resistance, toxicity, and unnecessary healthcare costs. The U.S. Centers for Disease Control and Prevention (CDC) strongly advocates for the inclusion of pharmacists as key leaders in the implementation and management of ASPs, given their expertise in pharmacotherapy and their critical role in influencing prescribing behaviors. Despite this recommendation, there remains a limited body of consolidated evidence evaluating the specific impact of pharmacist-led audit and feedback interventions within ASPs on antibiotic utilization. Furthermore, the mechanisms through which pharmacists contribute to improved prescribing—particularly via audit and feedback strategies—are not yet fully understood. This gap underscores the need for high-quality, systematic evaluation to determine the effectiveness of such interventions and to guide future integration of pharmacists into antimicrobial stewardship efforts [8,9].

This protocol forms the methodological foundation for a forthcoming systematic literature review and meta-analysis aimed at evaluating the impact of pharmacist-led audit and feedback interventions within ASP. By establishing a transparent and structured approach in advance, this protocol ensures methodological rigor, minimizes the risk of bias, and enhances the reproducibility of the review. The primary objective is to synthesize current evidence on how pharmacist-led audit-feedback influences antibiotic prescribing practices, with the broader goal of informing policy, clinical decision-making, and future research in antimicrobial stewardship.

## Description of protocol

### Protocol registration

To ensure transparency and methodological rigor in the conduct of this systematic review and meta-analysis, a protocol summary has been registered with the International Prospective Register of Systematic Reviews (PROSPERO) under the registration number CRD420251036088. The review will be conducted in accordance with the Preferred Reporting Items for Systematic Reviews and Meta-Analyses (PRISMA) guidelines [1]. A graphical abstract is included to visually summarize the methodological framework of the study.

### Research question

The research question guiding this systematic review and meta-analysis will be structured using the PICO framework (Population, Intervention, Comparison, and Outcome), which provides a clear and systematic approach for defining the key elements of the review. The components of the PICO framework applied in this study are as follows:

• Patient: Patient on antibiotics
• Intervention: Integration of audit and feedback intervention for antibiotic prescribing with pharmacist involvement
• Comparison: Without audit and feedback intervention for antibiotic prescribing with pharmacist involvement
• Outcome: Primary outcome: Days of therapy per (100/1000) patient days. Secondary outcome: appropriate prescribing, mortality, antibiotic resistance, infection recurrence rates, or length of hospital stay.

### Search strategy

A systematic literature search will be conducted across multiple electronic databases, including PubMed, Scopus, EBSCO/CINAHL, and Web of Science, to identify relevant peer-reviewed studies. The search will be restricted to publications from January 1, 2000, to June 31, 2024. A combination of predefined keywords and Boolean operators (e.g., “AND,” “OR”) will be employed to optimize the retrieval of pertinent literature. The full search strategy is detailed in Supplementary Material 1.

### Inclusion and exclusion criteria

Studies published in English that assess the impact of pharmacist involvement in antibiotic stewardship audit and feedback interventions will be included, based on the eligibility criteria outlined in Table 1.

**Table 1:** Inclusion and exclusion criteria.

Inclusion criteria:
1. English language 2. Studies in humans 3. Adult and pediatrics 4. Full text and data could be retrieved 5. Study type (randomized control trials, cohort studies, case-control studies, quasi-experiment studies) 6. Intervention includes antibiotic stewardship audit-feedback or equivalent with pharmacist involvement 7. Comparison / control group without pharmacist involvement 8. Measures include Days of therapy (DOT) per (100/1000) patient days, or appropriate prescribing or mortality or antibiotic resistance 9. Time frame (1 January 2000 – 30 June 2024) 10. Low or moderate risk of bias#
Exclusion criteria:
1. Studies in other languages 2. Studies in animals and environment 3. Review articles, protocols, qualitative studies, editorials, case reports, case series, expert opinion, conference proceeding/abstracts, conference review, book, book chapter, letter, erratum, note 4. Studies with multiple ASP intervention 5. Studies with high/critical high risk of bias# 6. Any other items contrary to the inclusion criteria

# Based on Cochrane risk of bias assessment, Δ pharmacist review of antibiotic order with intervention

### Data screening and extraction

**Fig. 1:**
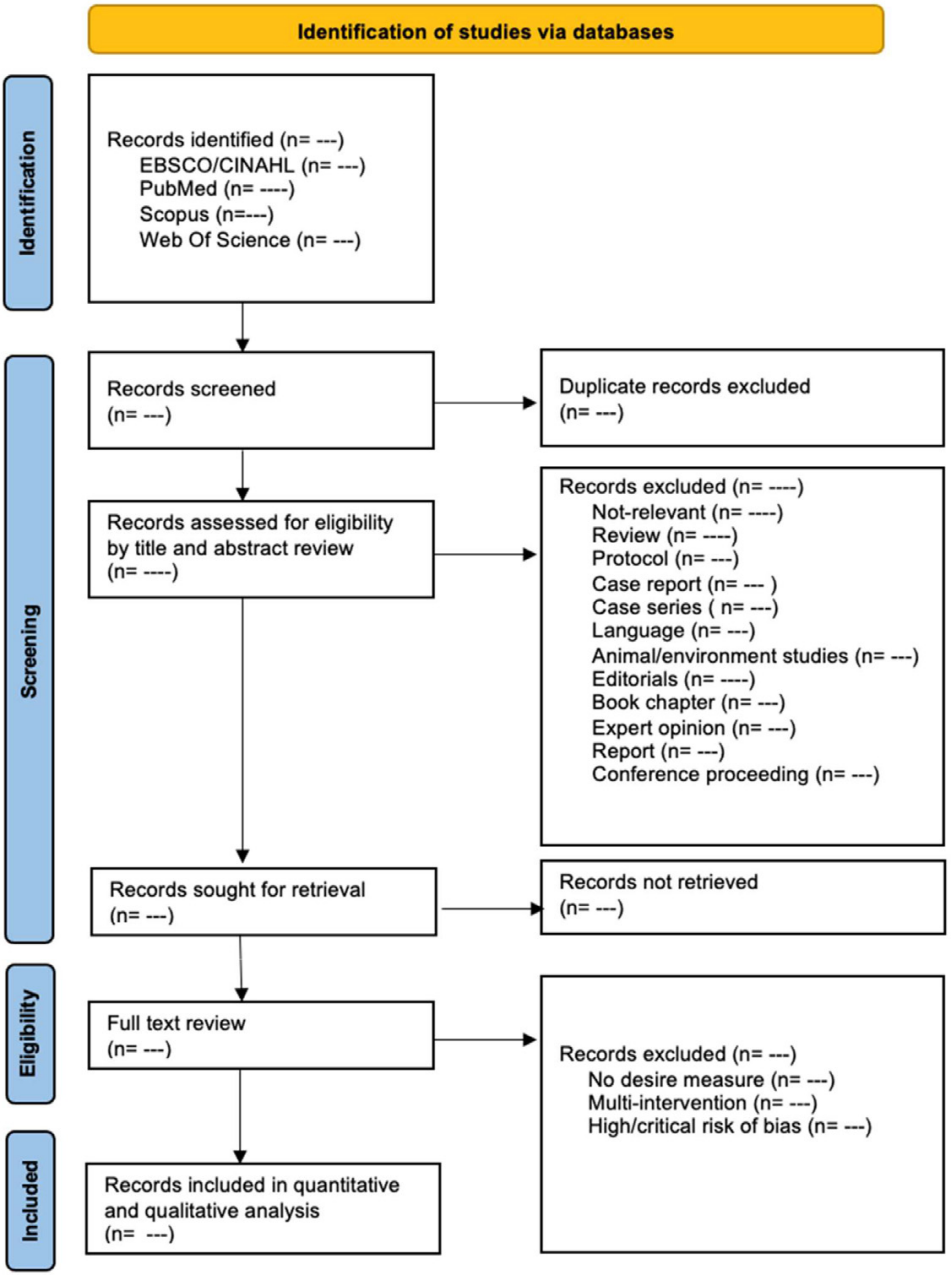
PRISMA flow diagram.

The screening process will be conducted in two stages. Initially, one reviewer will screen the titles and abstracts of all retrieved records, followed by full-text assessment for potentially eligible studies. A second reviewer will independently verify the screening and eligibility decisions. Any discrepancies between the reviewers will be resolved through discussion or consultation with a third reviewer, if necessary. All records retrieved from the databases will be imported into EndNote 21 reference management software for the identification and removal of duplicates. A manual verification will also be performed to ensure the thorough elimination of duplicate entries. The overall study selection process will be documented using a PRISMA flow diagram (Fig. 1). Data from all eligible studies will be extracted and managed using Microsoft Excel (16.95.1).

### Quality assessment

The risk of bias for included studies will be assessed using Cochrane tools based on study design. For cohort studies, case-control studies, and quasi-experimental designs, the Risk Of Bias In Non-randomized Studies of Interventions (ROBINS-I V2) tool will be applied. For randomized controlled trials, the Risk of Bias (RoB 2) tool will be used. The ROBINS-I tool evaluates risk of bias across seven key domains: confounding, classification of interventions, selection of participants, deviations from intended interventions, missing data, outcome measurement, and selection of the reported result. Each domain is assessed through a structured set of signalling questions designed to elicit detailed information about the study and its analytical approach. Once all relevant questions are answered, a predefined algorithm integrates the responses to generate an overall risk of bias judgement for each domain. These judgements are categorized as low, moderate, serious, or critical risk of bias, reflecting the extent to which bias may affect the study’s results. The RoB 2 tool offers a structured framework for assessing the risk of bias in the results of randomized trials. It encompasses five domains that address potential sources of bias arising from: the randomization process, deviations from the intended interventions, missing outcome data, measurement of the outcome, and selection of the reported result. Each domain includes a series of signalling questions designed to guide reviewers in evaluating the trial’s methodological rigor and to inform domain-level and overall risk of bias judgments. To visually present the risk of bias assessments, traffic-light plots will be generated using the Cochrane Risk-of-Bias Visualization (Robvis) tool [2,10,3,4]. Publication bias will be assessed through visual inspection of funnel plots, complemented by Egger’s regression test. A p-value of less than 0.05 will be considered indicative of significant publication bias.

### Data analysis

A qualitative synthesis will be undertaken to systematically organize and summarize key characteristics of the included studies. This synthesis will capture information such as author, year of publication, study design, setting, population, scope, intervention characteristics (e.g delivary mode, frequency, duration), Feedback team characteristics (e.g, specialty, experience), sample size, and measure (e.g days of therapy, length of hospital stay, mortality).

For the quantitative analysis, a random-effects meta-analysis model will be applied using R software (version 4.4.1) and RStudio (version 2024.09.0+375). To ensure comparability across studies, antibiotic use will be standardized as days of therapy per 100 patient-days (DOT/100 patient-days). Statistical heterogeneity among the included studies will be assessed using the I² statistic and interpreted in accordance with the guidelines provided in the Cochrane Handbook for Systematic Reviews of Interventions. Heterogeneity will be categorized as follows: low (<40%), moderate (30%–60%), substantial (50%–90%), and considerable (>75%). These overlapping thresholds reflect the contextual nature of heterogeneity interpretation, considering both the magnitude and consistency of effects across studies. In the presence of substantial heterogeneity, subgroup analyses will be conducted to explore potential sources of variability. These analyses will stratify the results based on study design, geographic region, and healthcare setting. Sensitivity analyses will be conducted using the leave-one-out method and prediction intervals to assess the robustness of the findings [5].

## Protocol validation

Not applicable.

## Limitations

A limitation of this study is that it focuses in the inclusion of only studies published in the English language. While this approach ensures consistency in data interpretation and quality assessment, it may introduce language bias and limit the comprehensiveness of the evidence base. Relevant studies published in other languages could have been excluded, potentially omitting valuable data and perspectives from non-English-speaking regions. As a result, the findings may not fully reflect global practices or outcomes related to pharmacist-led antibiotic stewardship interventions.

## CRediT author statement

**Duaa Jawhar:** Conceptualization, Methodology, Software, Writing- Original draft preparation. **Amer Khan:** supervision, validity, visualization, investigation, Writing- Reviewing and Editing. **Khorshid Alam:** Conceptualization, Methodology, Writing- Reviewing and Editing.

## Funds

This research did not receive any specific grant from funding agencies in the public, commercial, or not-for-profit sectors.

## Declaration of competing interest

The authors declare that they have no known competing financial interests or personal relationships that could have appeared to influence the work reported in this paper.

## Data Availability

Data will be made available on request.

## Appendix Supplementary materials

Image, application 1
