# Supplementary material for: Systematic review and meta-analysis protocol of impact of pharmacist-led antibiotic stewardship audit-feedback intervention
Source: MethodsX. 2025 May 30;14:103399. doi: 10.1016/j.mex.2025.103399 (PMC12177175; doi:10.1016/j.mex.2025.103399)
Supplement: Supplementary file 1 [file mmc1.docx]

**Supplementary Materials**

**Systematic review and meta-analysis protocol of impact of Pharmacist-Led Antibiotic Stewardship Audit-Feedback Intervention**

Table of Contents

[Table S1: PubMed Search string 1](#_Toc198976122)

[Table S2: Scopus, Web of Science, EBSCO/CINAHL Search string 2](#_Toc198976123)

[Table S3: PubMed search strategy 2](#_Toc198976124)

[Table S4: Scopus search strategy 3](#_Toc198976125)

[Table S5: Web of Science search strategy 4](#_Toc198976126)

[Table S6: EBSCO/CINAHL search strategy 5](#_Toc198976127)

[Table S7: Characteristics of included studies 6](#_Toc198976128)

# Table S1: PubMed Search string

| “Anti-Bacterial Agents*”[MeSH Terms] |
| --- |
| AND |
| (“audit feedback*” OR feedback* OR audit* OR intervention*) |
| AND |
| ( impact* OR effect* OR influence* OR evaluate* OR assess* ) |

# Table S2: Scopus, Web of Science, EBSCO/CINAHL Search string

| (Anti-Bacterial OR Antibiotic) |
| --- |
| AND |
| (audit feedback OR feedback OR audit OR Intervention) |
| AND |
| ( impact OR effect OR influence OR evaluate OR assess ) |

# Table S3: PubMed search strategy

| Search ID | Search description | Filter |
| --- | --- | --- |
| #8 | "Anti-Bacterial Agents*"[MeSH Terms] AND ("audit feedback*" OR feedback* OR audit* OR intervention*) AND ( impact* OR effect* OR influence* OR evaluate* OR assess* ) | from2000/1/1 - 2024/6/30 |
| #7 | ("Anti-Bacterial Agents*"[MeSH Terms]) AND ("audit feedback*" OR feedback* OR audit* OR intervention*) | from 2000/1/1 - 2024/6/30 |
| #6 | ("Anti-Bacterial Agents*"[MeSH Terms]) AND ("audit feedback*" OR feedback* OR audit* OR intervention*) |  |
| #5 | intervention* |  |
| #4 | audit* |  |
| #3 | feedback* |  |
| #2 | "audit feedback*" |  |
| #1 | "Anti-Bacterial Agents*"[MeSH Terms] |  |

# Table S4: Scopus search strategy

| Search  ID | | Search description | Filter |
| --- | --- | --- | --- |
| #7 | TITLE-ABS-KEY ( ( antibiotic OR anti-bacterial ) AND ( audit-feedback OR audit OR feedback OR intervention ) AND ( impact OR effect OR influence OR evaluate OR assess ) ) | | PUBYEAR > 1999 AND PUBYEAR < 2025 |
| #6 | TITLE-ABS-KEY ( intervention ) | |  |
| #5 | TITLE-ABS-KEY ( audit-feedback ) | |  |
| #4 | TITLE-ABS-KEY ( feedback ) | |  |
| #3 | TITLE-ABS-KEY ( audit ) | |  |
| #2 | TITLE-ABS-KEY ( antibacterial ) | |  |
| #1 | TITLE-ABS-KEY ( antibiotic ) | |  |

# Table S5: Web of Science search strategy

| Search ID | Search description | Filter |
| --- | --- | --- |
| #9 | ((((TS=(antibiotic )) OR TS=(anti-bacterial))) AND TS=((audit OR feedback OR audit-feedback OR intervention))) AND TS=(( impact OR effect OR influence OR evaluate OR assess )) | 2024 or 2023 or 2022 or 2021 or 2020 or 2019 or 2018 or 2017 or 2016 or 2015 or 2014 or 2013 or 2012 or 2011 or 2010 or 2009 or 2008 or 2007 or 2006 or 2005 or 2004 or 2003 or 2002 or 2001 or 2000 |
| #8 | ((((TS=(antibiotic )) OR TS=(anti-bacterial))) AND TS=((audit OR feedback OR audit-feedback OR intervention))) AND TS=(( impact OR effect OR influence OR evaluate OR assess )) |  |
| #7 | (((TS=(antibiotic )) OR TS=(anti-bacterial))) AND TS=((audit OR feedback OR audit-feedback OR intervention)) |  |
| #6 | TS=(intervention) |  |
| #5 | TS=(audit-feedback) |  |
| #4 | TS=(feedback) |  |
| #3 | TS=(audit) |  |
| #2 | TS=(anti-bacterial) |  |
| #1 | TS=(antibiotic) |  |

# Table S6: EBSCO/CINAHL search strategy

| Search  ID | | Search description | Filter |
| --- | --- | --- | --- |
| #8 | (Anti-Bacterial OR Antibiotic) ) AND ( (audit feedback OR feedback OR audit OR Intervention) ) AND ( ( impact OR effect OR influence OR evaluate OR assess ) | | 2000-2024 |
| #7 | (Anti-Bacterial OR Antibiotic) ) AND ( (audit feedback OR feedback OR audit OR  Intervention) ) AND ( ( impact OR effect OR influence OR evaluate OR assess ) | |  |
| #6 | Intervention | |  |
| #5 | Audit-feedback | |  |
| #4 | Feedback | |  |
| #3 | Audit | |  |
| #2 | Anti-bacterial | |  |
| #1 | Antibiotic | |  |

# Table S7: Characteristics of included studies

| Author, year | Country, region | Design | Setting | Population | Scope | Intervention. (e.g delivery mode, frequency, duration) | Feedback team characteristics (e.g, specialty, experience), | Sample size | Measure  (e.g days of therapy, length of hospital stay, mortality). |
| --- | --- | --- | --- | --- | --- | --- | --- | --- | --- |
|  |  |  |  |  |  |  |  |  |  |
|  |  |  |  |  |  |  |  |  |  |
